# Water assisted atomic layer deposition of yttrium oxide using tris(*N*,*N*′-diisopropyl-2-dimethylamido-guanidinato) yttrium(iii): process development, film characterization and functional properties†

**DOI:** 10.1039/c7ra13417g

**Published:** 2018-01-29

**Authors:** Lukas Mai, Nils Boysen, Ersoy Subaşı, Teresa de los Arcos, Detlef Rogalla, Guido Grundmeier, Claudia Bock, Hong-Liang Lu, Anjana Devi

**Affiliations:** Inorganic Materials Chemistry, Ruhr-University Bochum 44801 Bochum Germany anjana.devi@rub.de; Werkstoffe und Nanoelektronik, Ruhr-Universität Bochum 44801 Bochum Germany; Macromolecular and Technical Chemistry, University of Paderborn 33098 Paderborn Germany; RUBION, Ruhr-University Bochum 44801 Bochum Germany; Mikrosystemtechnik, Ruhr-Universität Bochum 44801 Bochum Germany; Institute of Advanced Nanodevices, School of Microelectronics, Fudan University Shanghai 200433 China

## Abstract

We report a new atomic layer deposition (ALD) process for yttrium oxide (Y_2_O_3_) thin films using tris(*N*,*N′*-diisopropyl-2-dimethylamido-guanidinato) yttrium(iii) [Y(DPDMG)_3_] which possesses an optimal reactivity towards water that enabled the growth of high quality thin films. Saturative behavior of the precursor and a constant growth rate of 1.1 Å per cycle confirm the characteristic self-limiting ALD growth in a temperature range from 175 °C to 250 °C. The polycrystalline films in the cubic phase are uniform and smooth with a root mean squared (RMS) roughness of 0.55 nm, while the O/Y ratio of 2.0 reveal oxygen rich layers with low carbon contaminations of around 2 at%. Optical properties determined *via* UV/Vis measurements revealed the direct optical band gap of 5.56 eV. The valuable intrinsic properties such as a high dielectric constant make Y_2_O_3_ a promising candidate in microelectronic applications. Thus the electrical characteristics of the ALD grown layers embedded in a metal insulator semiconductor (MIS) capacitor structure were determined which resulted in a dielectric permittivity of 11, low leakage current density (≈10^−7^ A cm^−2^ at 2 MV cm^−1^) and high electrical breakdown fields (4.0–7.5 MV cm^−1^). These promising results demonstrate the potential of the new and simple Y_2_O_3_ ALD process for gate oxide applications.

## Abstract 2

A new water assisted atomic layer deposition (ALD) process was developed using the yttrium tris-guanidinate precursor which resulted in device quality thin films.

## Graphical abstract

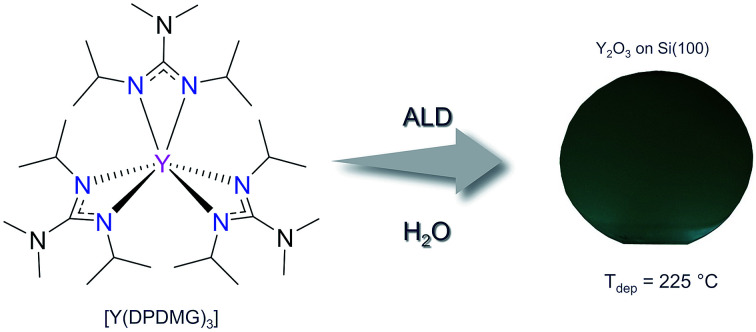

## Introduction

**Scheme 1:**
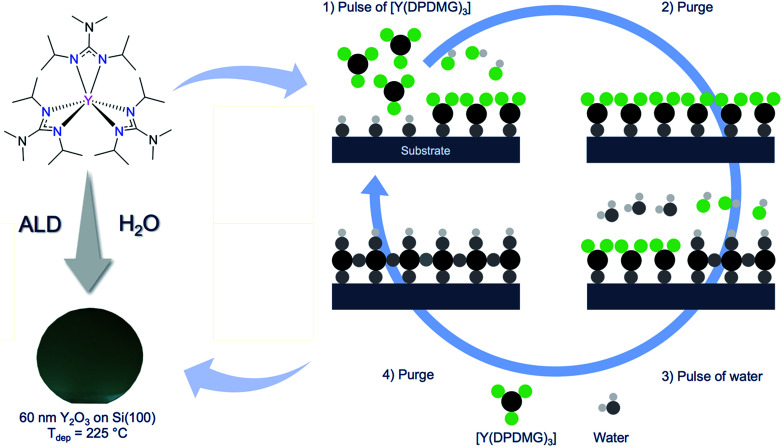
Molecular structure of [Y(DPDMG)_3_]^24^ (top-left). Highlighted are the six reactive Y–N bonds. (Bottom-left) A photograph of a 50 nm thick Y_2_O_3_ thin film grown on 2-inch Si(100) wafer at 225 °C. (Right) A typical schematic representation of an ALD cycle involving the two highlighted precursors.

Yttrium(iii) oxide (Y_2_O_3_) exhibits beneficial intrinsic properties, that render this material exceptionally useful to be implemented in modern devices, such as micro- and optoelectronics. The high refractive index of *n* = 2.1 was especially useful for the fabrication of planar waveguides in solid state and high power lasers.^1–3^ Moreover, a high thermal conductivity of 0.27 W (cm K)^−1^ at 300 K,^4^ a high melting point of 2430 °C and a high mechanical strength make this material conducive for other solid state applications,^5,6^ such as temperature and wear resistive coatings and hydrophobic house hold coatings.^7^ To meet the demands for smaller, yet more effective transistors, the thickness of the functional layers in these transistors has to shrink. To retain the performances of the transistor and hinder tunnelling-effects in the thin dielectric material, high-*κ* gate dielectrics must be employed. Thin films of Y_2_O_3_ are suited as high-*κ* gate dielectrics due to the large intrinsic band gap in the range of 5.5–5.8 eV and high dielectric constant of *κ* = 14–18. For this reason, Y_2_O_3_ thin films were intensively studied in metal oxide semiconductor field effect transistors (MOSFETs) as the thin high-*κ* gate material.^8–10^ As such microelectronic devices are often very sensitive to high temperatures, the nano-scaled thin films have to be dense, conformal, uniform and should be deposited at low temperatures. Atomic layer deposition (ALD) encompasses all the mentioned features and thus, is an indispensable method for some aspects such as gate oxide deposition in the microelectronic industry today, not least because of the possibility to precisely control the thickness at an atomic level.^11–13^ An ideal ALD process is defined by three distinct characteristics: first, the growth rate should be independent from the precursor pulse time once the saturation of the surface is reached. Second, the film thickness must be in linear dependence to the number of the applied ALD cycles. Third, in most ALD processes saturation and linear behaviour is observed within a defined temperature regime in which the growth per cycle (GPC) is independent from the deposition temperature (ALD window). For all these features to be realized, the employed chemistry (including the co-reactants) plays a pivotal role. Thus, the right choice of the precursors for the development of an ALD process is of great importance, especially with respect to the reactivity and thermal stability. Several precursors for the ALD of Y_2_O_3_ have been reported and among them the most prominent yttrium precursor for ALD can be assigned to the group of β-diketonates, namely [Y(thd)_3_], (thd = 2,2,6,6-tetramethyl-3,5-heptanedione). Because of the Y–O bonds within this complex, the reactivity towards oxygen functionalities on the surface is very limited. Thus, a strong oxidizing agent such as ozone had to be used as reactive co-reactant at high deposition temperatures of 350 °C which yielded growth rates of only 0.22 Å per cycle.^14^ Even β-diketonates paired with organic adducts (bipyridyl or 1,10-phenanthroline) in [Y(thd_3_)(bipy)] and [Y(thd)_3_(phen)] did not have an impact on the growth behaviour. On the contrary, the use of reactive yttrium cyclopentadienyls with different ligand substitution patterns, such as [Y(Cp)_3_], [Y(MeCp)_3_], [Y(EtCp)_3_] and [Y(iPrCp)_3_] allowed the use of water as a mild oxygen source.^15–17^ The growth rates reported were higher and ranged between 1.2 Å per cycle for [Y(MeCp)_3_], 1.6 Å per cycle for [Y(Cp)_3_] and 1.7 Å per cycle for [Y(EtCp)_3_] and [Y(iPrCp)_3_]. The ALD window involving [Y(Cp)_3_] or [Y(MeCp)_3_] as reactive precursors range from 250 °C to 300 °C, while only at higher temperatures the contamination with C and H are reduced in the thin films. The other ALD process involving [Y(EtCp)_3_], only possesses a narrow ALD window in the range of 250–285 °C, while stoichiometric and films with low contamination levels were obtained. In a different study, the homoleptic yttrium tris(*N*,*N*′-diisopropylacetamidinate) [Y(iPr-amd)_3_] and water as an oxygen source was used by Gordon *et al.*^18^ Due to the high oxophilicity of yttrium, high water purge times have been used, which resulted in long deposition sequences. A constant growth rate of 0.9 Å per cycle in the temperature range 150 °C to 280 °C yielded Y_2_O_3_ thin films. Recently, a heteroleptic liquid yttrium precursor, which consists of cyclopentadienyl and amidinate ligands [Y(iPrCp)_2_(iPr-amd)] together with water, was reported for the fabrication of stoichiometric Y_2_O_3_ thin films by Lansalot-Matras *et al.*^19^ In this case, the ALD-window was found at high temperatures ranging from 350–450 °C and growth rates in the order of 0.6 Å per cycle were achieved. In the past, our research group developed efficient ALD processes for lanthanide oxides such as Gd_2_O_3_, Dy_2_O_3_ and Er_2_O_3_, using the homoleptic tris-guanidinates ([Ln(DPDMG)_3_], where Ln = Gd, Dy, Er) in a simple water assisted process resulting in device quality layers.^20,21^ The analogous yttrium compound [Y(DPDMG)_3_] was successfully employed for the metalorganic chemical vapour deposition (MOCVD) of Y_2_O_3_ thin films but wasn't evaluated for ALD applications.^22^ This compound possesses high reactivity, chemical and outstanding thermal stability making it suitable for ALD applications. The presence of the six Y–N-bonds (Scheme 1) within this complex render the complex highly reactive toward oxygen functionalities, making it plausible for a water assisted ALD process for Y_2_O_3_. The high reactivity originates from a strong oxophilicity of the respective rare-earth metal.^23^ This has been successfully demonstrated earlier for other lanthanide oxide (Gd_2_O_3_, Dy_2_O_3_, Er_2_O_3_) ALD processes using the homoleptic tris-guanidinate precursors.^20,21^ We build upon our recent advances in new ALD process development and here in we report a new water assisted ALD process for Y_2_O_3_ thin films. The ALD characteristics were verified and the deposited thin films were characterized with respect to their crystallinity, morphology and composition. Furthermore, the optical properties were investigated and first investigations on the application of Y_2_O_3_ as gate oxides in MIS capacitors were performed.

## Experimental section

The homoleptic yttrium tris-guanidinate precursor [Y(DPDMG)_3_] was synthesized in an up-scaled synthesis (10 g) following a previously published procedure by our group.^24^

The thermal ALD of Y_2_O_3_ was carried out in a commercial F-120 reactor (ASM Microchemistry Ltd.) at 0.1–2 mbar on 2′′ Si(100) wafers with 200 mg of [Y(DPDMG)_3_] as precursor vaporized at 130 °C and water (HPLC grade) as co-reactant maintained at room temperature. The substrates were cleaned with HPLC grade isopropanol and water and ultrasonicated in water for 30 min. After cleaning, the wafers were dried under a nitrogen gas flow. Nitrogen (AirLiquide, 99.9999%) was used as carrier and purging gas. The following optimized pulse/purge sequence was used during deposition: 4 s [Y(DPDMG)_3_] pulse, 20 s purge, 3 s water pulse and 30 s purge in a temperature range of 150–275 °C. Film thickness was determined *via* X-ray reflectometry (XRR; Bruker D8 Discover XRD) with Cu-Kα radiation (1.5418 Å) in a *Θ*–2*Θ* locked coupled mode, while 2*Θ* was increased from 0.1° to 3° with a step size of 0.01. Grazing incidence X-ray diffraction was carried out using a PANalytical X'pert pro diffractometer. Rutherford backscattering spectrometric (RBS) analysis and nuclear reaction analysis (NRA) were performed at the RUBION, the Central Unit for Ion Beams and Radionuclides at Ruhr-University Bochum. For RBS, a 2.0 MeV ^4^He^+^ ion beam with an intensity of 20–40 nA was directed to a sample with an angle of 7°. The scattered particles were detected by a Si detector with a resolution of 16 keV at 160°. NRA was performed to obtain the concentration of elements with a low atomic number, like C, N and O. The concentration was obtained after an induced nuclear reaction of the light elements by a 1.0 MeV deuteron beam and detection of the emitted protons at 135°. A 6 μm Ni foil was used to shield the detector from scattered deuterons. The beam penetrates the whole thin film including the sample. The software suite SimNRA was used to estimate the concentration of the elements in the thin film, by using the data obtained by the RBS and NRA measurements.^25^ X-ray photoelectron spectroscopy (XPS) analysis of the yttrium(iii) oxide thin films were conducted at the University of Paderborn (UPB). XPS was performed using an Omicron ESCA+ system (Omicron NanoTechnology GmbH) equipped with a hemispherical energy analyzer at a base pressure of <5 × 10^−10^ mbar. Spectra were recorded at a pass energy of 20 eV, leading to a full-width half maximum of the Ag 3d_5/2_ peak of 0.77 eV. A monochromatic Al Kα (1 486.7 eV) X-ray source was used, with a spot diameter of 1 mm. The spectra were measured under a take-off angle of 45°, and under irradiation with a low energy electron beam for charge compensation (4.1 eV energy, 5 μA sample current). It should be noted, that only the surface is analysed with this technique with a penetration depth of approx. 5 nm. For the peak analysis, a convolution of Gaussian and Lorentzian line shapes was used after subtraction of a Shirley background. The Gaussian components were left free in the fit; the width of the Lorentzian components was fixed to 0.10 eV for the Y 3d peak and 0.13 eV for the O 1s peak. The binding energy (BE) scale was determined by fixing the position of the C 1s peak from adventitious carbon to 284.7 eV. Where indicated, removal of the superficial film layers was done by sputtering with Ar^+^ ions with 2 keV energy for 2 minutes. For the peak analysis, a convolution of Gaussian and Lorentzian line shapes was used after subtraction of a Shirley background. Atomic force microscopy (AFM) measurements were performed using a Nanoscope Multimode V microscope from Digital Instruments, operating in tapping mode. UV/Vis measurements of 30 nm thin films deposited on fused silica substrates were carried out using a double beam spectrophotometer (Agilent Cary 5000). Electrical characterisation of the samples was carried out on metal–insulator–semiconductor (MIS) capacitors. For this 20 nm Y_2_O_3_ was deposited onto a n^+^-type Si(100) substrate. Ti/Au (3 nm/70 nm) gate electrodes with a diameter of 50 μm were e-beam evaporated onto the Y_2_O_3_ film surface through a shadow mask. To extract the permittivity of the dielectric material, capacitance–voltage (*C*–*V*) measurements were performed using an Agilent E4282 A LCR meter. For the current–voltage (*I*–*V*) characteristics of the MIS structures a semiconductor parameter analyzer (Agilent 4156B) was used.

## Results and discussion

### ALD process optimization

**Fig. 1:**
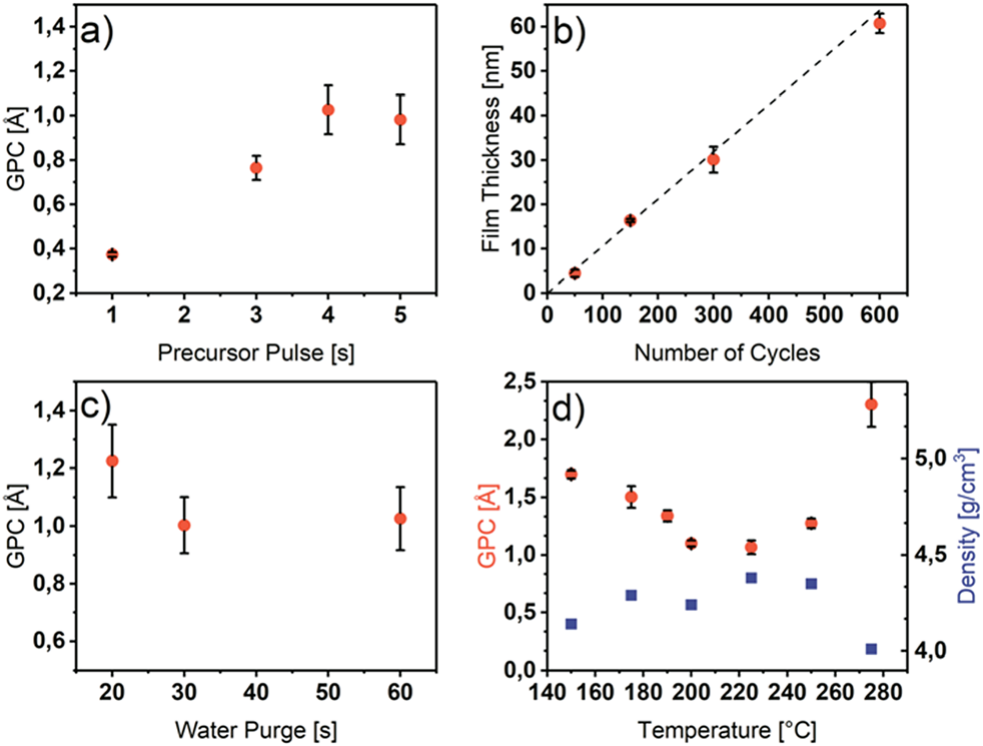
(a) Precursor saturation studies by the variation of the [Y(DPDMG)_3_] pulse length at 225 °C on Si(100). (b) Film thickness *versus* number of applied cycles. (c) GPC variation with water purge time at 225 °C on Si(100). (d) GPC and thin film density as a function of deposition temperature.

Since the uniformity, conformity and precisely tunable thickness of ALD thin films is strongly dependent on the self-limiting reactions of the process, a process optimization with respect to the ALD characteristics is necessary. To optimize the ALD process, in order to obtain uniform, dense and high quality Y_2_O_3_ thin films with high growth rates, a series of depositions were performed in which process parameters like the precursor pulse/purge times, the deposition temperature and the number of ALD cycles were varied systematically. The most important characteristic to prove self-limiting ALD behavior is the saturation growth as a function of the precursor pulse length in a defined temperature range. As shown in Fig. 1a, for the saturation study, the precursor pulse length was varied from one to five seconds, while the other parameters such as the deposition temperature (225 °C), precursor purging time (20 s), water pulse length (3 s) and water purging time (30 s) were kept constant.

As can be seen in Fig. 1a, the precursor saturates the surface after 4 s with a constant GPC of 1.1 Å. For longer pulse times, no higher growth rates were obtained if a water purge time of 30 s was applied to ensure a sufficient removal of adsorbed water species (Fig. 1c). Below 30 s of water purge time, an increased growth rate is observed possibly due to a reaction of additional adsorbed water molecules on the surface hydroxyl groups with the [Y(DPDMG)_3_] precursor. This effect was also observed previously by R. Gordon *et al.* for the ALD of Y_2_O_3_ with the homoleptic yttrium tris-amidinate precursor and water.^18^ However, the Y_2_O_3_ surface of this process seems to be even more hydrophilic since a water purge time of 60 s was needed for a self-limiting growth.

In order to identify the temperature range of self-limiting growth, the substrate temperature was varied from 150 °C to 280 °C. An ALD window ranging from 175 °C to 250 °C with a GPC between 1.1 Å and 1.3 Å could be observed. Furthermore, within the ALD window, the density of the resulting films was found to be between 4.2 g cm^−3^ and 4.4 g cm^−3^, while outside the window it decreases to 4.0 g cm^−3^.

Thus, below 175 °C the precursor most likely condenses on the substrate, which is corroborated by a higher GPC, a lower density (Fig. 1d) and the composition of the films (discussed later, Table 1). At 275 °C, the precursor tends to decompose which was proven with differential thermal analysis (DTA), in which the decomposition temperature was found to be 267 °C (Fig. S2†). This decomposition leads to parasitic CVD growth and thus, a strongly increased GPC, a lower density as evidenced in Fig. 1d as well as a non-uniform growth.

**Table 1:** Compositional analysis as a function of deposition temperature as determined from RBS/NRA

*T* [°C]	O/Y ratio	Yttrium [at%]	Oxygen [at%]	Nitrogen [at%]	Carbon [at%]
150	2.2	29	64	2	5
175	2.1	31	64	2	3
200	2.1	31	64	2	3
225	2.0	32	64	3	2
250	2.1	31	66	2	2
275	2.3	29	66	1	5

The variation of the film thickness as a function of the number of applied ALD cycles was also investigated. As illustrated in Fig. 1b, the thickness increases linearly with the number of applied cycles. With a linear fit of the measured film thicknesses at different number of applied cycles, an overall GPC of 1.06 Å could be determined. The low error values, indicated by a *R*^2^ value of 0.998 of the fit shows, that the thickness indeed is precisely tunable with our optimized process.

### Thin film characterization

#### Film crystallinity

To evaluate the crystallinity of a Y_2_O_3_ thin film deposited at 200 °C (30 nm), grazing incidence X-ray diffraction (GI-XRD) was carried out. The as-deposited film is polycrystalline in the cubic phase of Y_2_O_3_ depicted by the characteristic (222), (400) and (440) reflexes (Fig. S4†). The low temperature onset of crystallization may be attributed to the low lattice mismatch between Si(100) and cubic Y_2_O_3_, as the lattice parameter of the cubic Y_2_O_3_ phase (*a* = 1.064 nm) closely matches the lattice parameter of Si(100) (2*a* = 1.086 nm).^26^ Moreover, the density of polycrystalline Y_2_O_3_ films deposited at 200 °C (4.24 g cm^−3^) is significantly lower as the single crystalline bulk of Y_2_O_3_ (5.03 g cm^−3^), which supports the idea of grain boundaries present in the polycrystalline Y_2_O_3_, that lower the density of the material.^27^

#### Thin film morphology

**Fig. 2:**
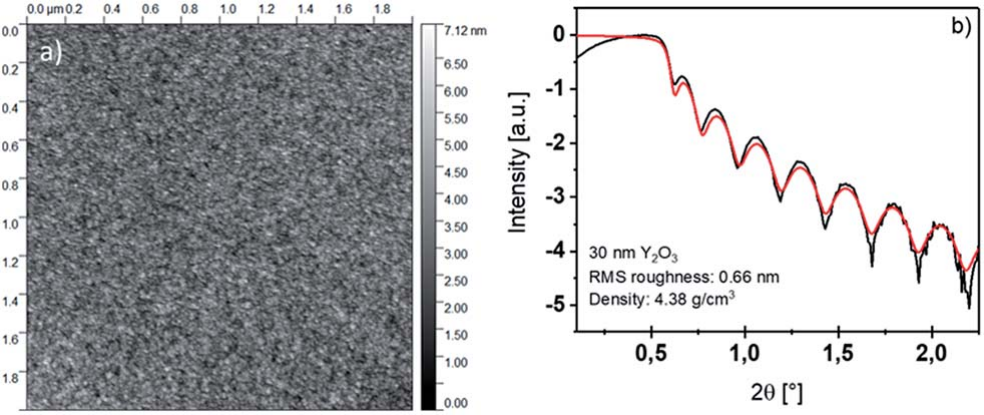
(Left) AFM image of a 2 × 2 μm spot derived from a 20 nm Y_2_O_3_ thin film deposited at 200 °C. (Right) X-ray reflectivity curve of a 30 nm Y_2_O_3_ thin film deposited at 225 °C. The measured curve is depicted in black, while the fitted curve is shown in red.

The thin film morphology was evaluated by AFM performed on a 20 nm Y_2_O_3_ film deposited under optimized conditions at 200 °C. As shown in Fig. 2a, the deposited thin film is smooth, as expected for an ALD-type growth, with a RMS roughness of 0.55 nm. The underlying substrate roughness prior to deposition was evaluated for a comparison with the roughness of the as deposited Y_2_O_3_ and was found to be 0.20 nm for the native SiO_2_ on a Si(100) substrate. The RMS roughness obtained by AFM is moreover supported by the results obtained *via* XRR measurements: The calculated roughness derived *via* this method is 0.66 nm for a 30 nm thin film deposited at 225 °C (Fig. 2b). The small deviation of the RMS roughness of 0.35 nm shows the high process control that can be achieved with a thoroughly optimized ALD process with [Y(DPDMG)_3_].

### Composition analysis

#### RBS/NRA

The composition of the Y_2_O_3_ thin films at different deposition temperatures was estimated by RBS and NRA. A representative RBS spectrum is shown in Fig. S3,† where the Y peak can be clearly observed. Overlapping with the silicon substrate signal, an oxygen peak is visible as well, while from NRA only minor carbon and nitrogen contaminations of the thin films were detected. In Table 1, the determined composition of the thin films grown at different deposition temperatures are summarized. Within the ALD window, the thin films are oxygen rich with an oxygen to yttrium ratio of 2 and low levels of contamination from C (∼2–3 at%) and N (∼2 at%). The carbon contamination of the thin films increases with lower temperatures and deviates between 2.0 at% (250 °C) and 3.4 at% (175 °C). For nitrogen the values vary between 1 at% at 275 °C and 3 at% at 225 °C, which is not unusual since the errors for the nitrogen content from NRA measurements in this low concentrations tend to be higher than for other elements.

Interestingly, for deposition temperatures outside the ALD window of 150 °C and 275 °C, the carbon contamination rises to 5 at%. This observation is in accordance with our assumption of precursor condensation at lower temperatures and decomposition at higher temperatures with respect to the ALD window temperature.

#### XPS

To gain more information about the chemical composition of the thin films and chemical binding behavior of the atoms within the thin film, XPS was performed. For a 30 nm thin film deposited at 225 °C, XPS revealed a Y_2_O_3_ film, that is oxygen rich at the surface and oxygen deficient after sputtering in the bulk of the material. Without sputtering, a high carbon and silicon contamination on the surface could be detected, which is most likely caused by adsorbed residuals because of the contact with ambient conditions. Moreover, the O/Y ratio of the thin film is 1.8, which is also in accordance with the ratio obtained by RBS/NRA measurements. In the bulk of the thin film, the ratio of O/Y decreases to 1.24, which is below the theoretical stoichiometric Y_2_O_3_ ratio of 1.5 (Table S1†). The lower O/Y ratio as expected for stoichiometric Y_2_O_3_ may be explained by preferential sputtering of the oxides by Ar^+^. The carbon contamination in the bulk of the thin film after sputtering was 2.6 at%, while nitrogen and silicon were below the detectable limit. These values are in accordance with contaminations obtained from RBS/NRA measurements, where the carbon contamination is within the same range as for the XPS measurement at around 2.5 at%. Here, we want to highlight the excellent reactivity of the [Y(DPDMG)_3_] precursor which seems to have a clean reaction with water to form Y_2_O_3_ with volatile by-products, resulting in only low carbon impurities.

**Fig. 3:**
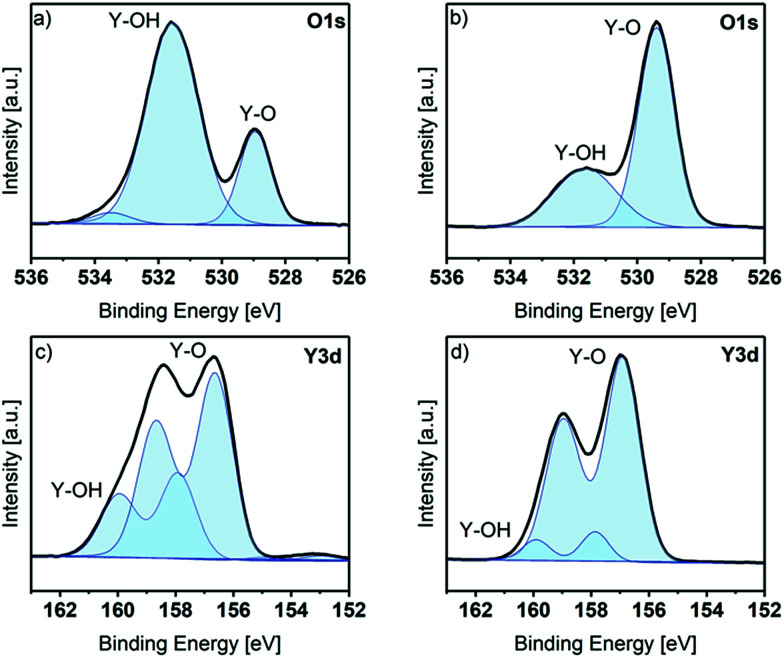
XPS O 1s and Y 3d core level spectra of the thin film deposited at 225 °C (30 nm). (a) Normalized O 1s peak as-introduced. (b) Normalized O 1s peak after sputtering. (c) Normalized Y 3d peak as-introduced. (d) Normalized Y 3d peak after sputtering. Blue curve: fitted regions for different core contributions. Black curve: measured data.

In order to get a more detailed look into the binding situation especially of Y and O, the core spectra of the as deposited and the sputtered samples were recorded. The Y 3d and O 1s core level peaks are shown in Fig. 3. Before sputtering, the O 1s peak shows three components at BE positions of 529.0 eV (assigned to Y–O bonds), 531.6 eV (assigned to Y–OH bonds) and 533.5 eV (assigned to adsorbed water species).^28–30^ The existence of Y–OH bonds and adsorbed water species is in accordance to the ALD process, which ended with a water pulse and purge and thus, reactive hydroxyls can remain on the surface (Fig. 3a). This observation was also proven with contact angle measurements: the contact angle of as deposited thin films was in a range of 10°, which corresponds to a strongly hydrophilic surface. The Y 3d core level peak shows two different contributions, identified by two doublets. The spin–orbit doublet components were fitted with a fixed separation of 2.05 eV between the 3d_5/2_ and 3d_3/2_ components, and an intensity ratio between 3d_3/2_ and 3d_5/2_ of about 0.7. The Y 3d_5/2_ component with BE at 156.7 eV is assigned to Y–O. A second component appears at 157.9 eV, which is assigned to Y–OH. A weak signal is also visible at around 153 eV, associated to the Si 2s peak of adsorbed silicon species (2.6 at% of Si was detected on the as-introduced sample), probably originating from adsorption of silicon grease during transfer of the sample to the spectrometer.^28,29,31^ The C 1s peak reveals three different contributions at 284.7 eV (carbon, C–H, major), 285.9 eV (C–OH, minor) and 289.2 eV (O–C=O, minor) as depicted in Fig. S5,† attributed to adventitious carbon (the carbon amount in the sample as-introduced was 35.4 at%).^32^

After sputtering, only Y, O and a small amount of C are present on the sample surface. The O 1s core level peak reveals only two different contributions at 529.1 eV and 531.3 eV, assigned as before to Y–O and Y–OH bonds respectively. Interestingly, the intensity of the contributions is the opposite to that which was explored before sputtering. The still detectable Y–OH bonds in the bulk of the film, although the contribution is only minor, might be caused by an insufficient coverage of all hydroxyl groups after the precursor pulse. This can be explained by the steric demand of the guanidinate backbone, which may shield some hydroxyl groups and protect them from other precursor molecules. The Y 3d peaks show only two components, at Y 3d_5/2_ positions of 156.6 and 157 eV, corresponding to Y–O and Y–OH. Here the lower intensity of the –OH component is also seen after sputtering. The C 1s core spectrum after sputtering (see Fig. S5†) reveals three different contributions at 284.6 eV (carbon, C–H, minor), 289.2 eV (O–C=O, minor) and 289.9 eV (O=C(–O)_2_, major). While these carbon impurities are detectable in the bulk of the film, they are minor and correspond to a concentration of 2.6 at% in the overall composition of the film.

### Functional properties

#### Optical characterization

**Fig. 4:**
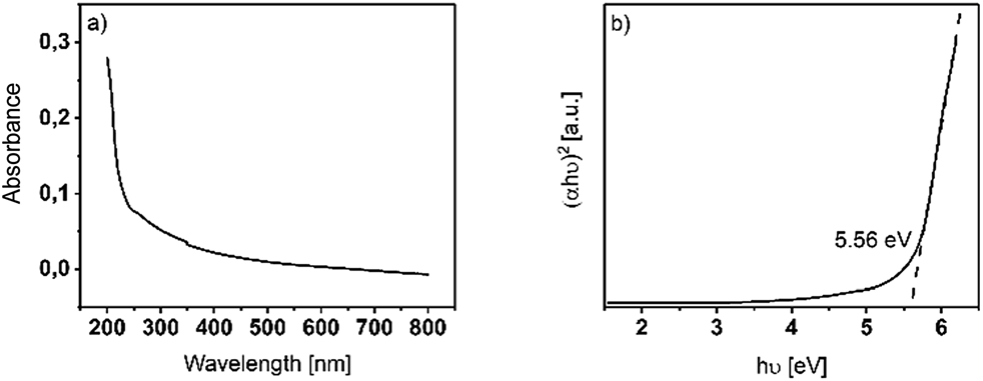
(a) Absorbance spectrum of a 20 nm thin Y_2_O_3_ film deposited at 225 °C on fused silica. (b) Tauc-plot representation of a 20 nm thin Y_2_O_3_ film deposited at 225 °C on fused silica.

Y_2_O_3_ can be used as high-*κ* gate oxide in MOSFETs and thus, the band gap is of high importance. Therefore, the optical properties were investigated by performing UV/Vis spectroscopy (Fig. 4a) to get an estimate of the band gap energy (*E*_g_) *via* a Tauc plot representation (Fig. 4b). For this, a 20 nm film of Y_2_O_3_ was grown at 225 °C on transparent fused silica substrates and the transmission was measured in the UV/Vis range of the light ranging from 200 nm to 800 nm. The 20 nm thick film absorbs the light up to 50% from 200 nm to 300 nm and tend to be transparent in the visible range of the light from 300 nm to 800 nm.

As for Y_2_O_3_, an allowed optical direct bandgap is reported, and we calculated the (*αhν*)^2^ term for such bandgaps in the Tauc representation from the UV-Vis spectrum. The bandgap could be determined by the intersection point of a linear extrapolation of the linear regime in the Tauc plot with the *X*-axis. The bandgap energy was found to be *E*_g_ = 5.56 eV, which is in accordance with literature reported optical bandgaps in the range of *E*_g_ = (5.5–5.8) eV.^8^

#### Electrical characterization

**Fig. 5:**
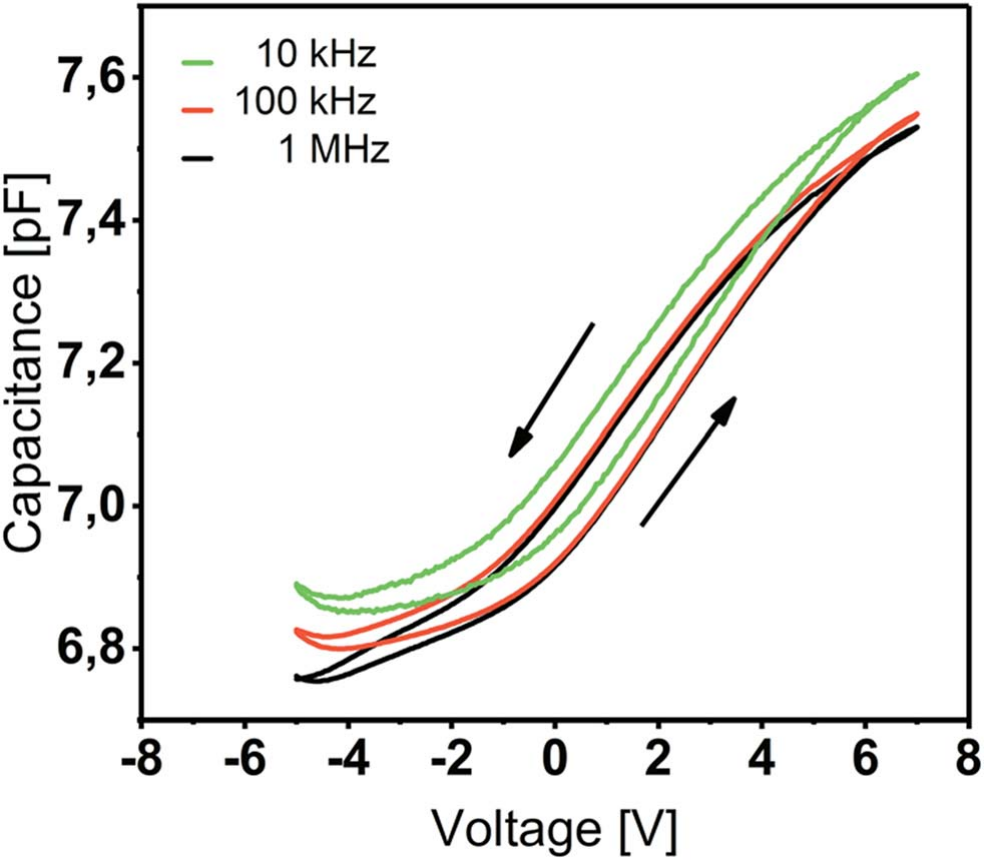
Capacitance–voltage curves of Y_2_O_3_ based MIS capacitors for *f* = 10 kHz, 100 kHz and 1 MHz. The 20 nm Y_2_O_3_ thin film was deposited at 200 °C.

The *C*–*V* and *I*–*V* characteristics of the ALD grown Y_2_O_3_ films were investigated in the form of MIS capacitors with Ti/Au top electrodes. The permittivity of the Y_2_O_3_ film (thickness = 20 nm) was calculated from *C*–*V* measurements, which are shown in Fig. 5 for Au/Ti/Y_2_O_3_/n^+^-Si(100) capacitors at frequencies *f* = 10 kHz, 100 kHz and 1 MHz. The permittivity is derived from the maximum capacitance in the accumulation regime, where the series capacitance of the depletion zone is negligible, and the measured value corresponds to the capacitance of the insulating layer. Taking into account a 2 nm thick native SiO_2_ film on top of our n^+^-Si substrate, we estimated a permittivity of 11.

This value is line with the data reported by other groups on atomic-layer deposited Y_2_O_3_.^15,16,18^ The *C*–*V* characteristics (Fig. 5) show anticlockwise hysteresis of about 1 V, which can be explained by the presence of trap states, *e.g.* rechargeable oxide traps. Flat-band voltages of 2.2–2.4 V indicate a non-negligible amount of negative fixed charge within the film.^33^ We assume an increased –OH content in the Y_2_O_3_ film which was proven by XPS studies and that occurs since no provisions were made to prevent water penetrating from the atmosphere after the ALD process.

**Fig. 6:**
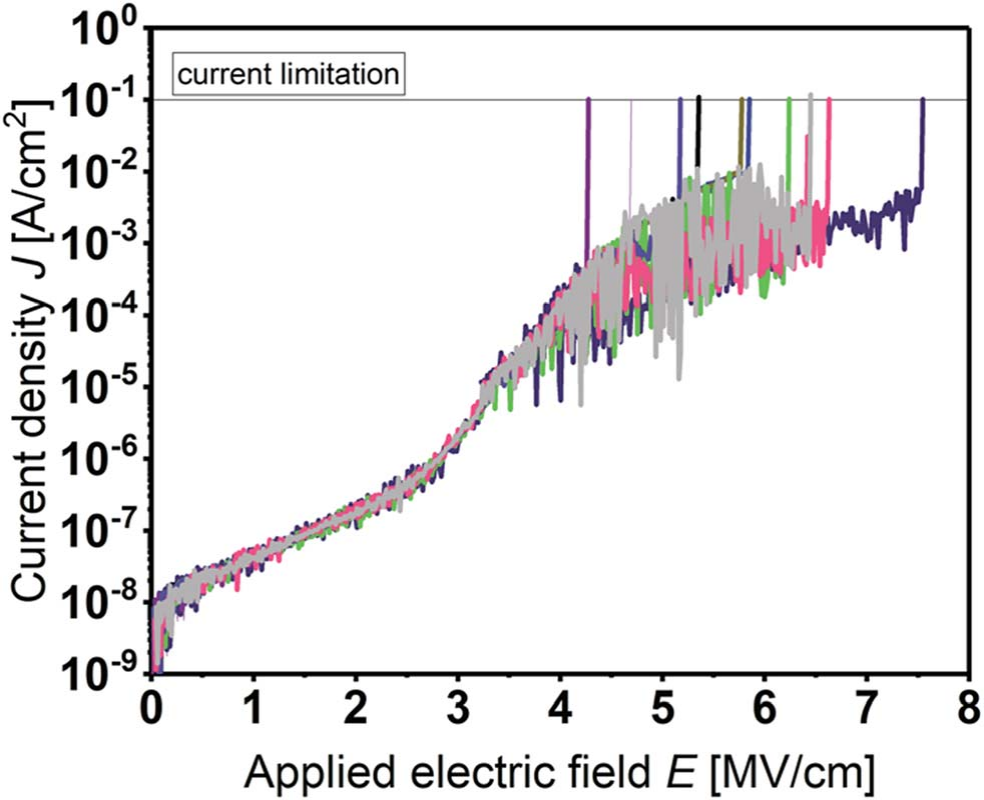
Leakage current density *J* as a function of the applied electric field *E* of several MIS capacitors incorporating Y_2_O_3_. Each color represents a *J*–*E* characteristic of an individual device with identical device geometries. The 20 nm Y_2_O_3_ thin film was deposited at 200 °C.

Prior to *C*–*V* studies, the leakage current of the MIS devices was measured. Fig. 6 shows the current density *J* as a function of the applied electrical field *E* for several devices. All films show a high breakdown field between 4.0 and 7.5 MV cm^−1^ and a low leakage current density of about 10^−7^ A cm^−2^ at 2 MV cm^−1^.

This makes the developed ALD process for Y_2_O_3_ attractive for the fabrication of a gate-dielectric in transistor structures and due to the low process temperatures even applicable on flexible polyimide foils.

## Conclusions

The application of yttrium tris-guanidinates for ALD of Y_2_O_3_ thin films is a first example and this was possible owing to the high reactivity of the all-nitrogen coordinated guanidinate ligands towards OH functionalities. Thus, we have successfully demonstrated a promising ALD process for Y_2_O_3_ which was solely water driven avoiding the use of strong oxidants like ozone generally used for metal oxides. This underlines the importance of identifying suitable precursors to be employed in an ALD process. The crystallinity of the Y_2_O_3_ films deposited at low temperatures and with low thickness can also be accounted for the high reactivity of the precursor towards water. Typical ALD characteristics in terms of ALD window, saturation behaviour, linear thickness dependence was confirmed under optimised process conditions, and the resulting Y_2_O_3_ films were homogeneous and smooth. From the electrical measurements on MIS capacitor structures, the obtained low leakage currents (10^−7^ A cm^−2^ at 2 MV cm^−1^), permittivity of 11 and high electric breakdown fields (4.0–7.5 MV cm^−1^) exemplify the high quality of the Y_2_O_3_ films obtained from the new ALD process developed in this study. Thus, this process is strikingly interesting for high-*κ* dielectrics in transistor structures. Due to their oxygen rich surface, thin films of Y_2_O_3_ deposited with this process could be envisaged as passivation layer in metal oxide thin film transistors (MOTFT), to enhance their stability and improve their electrical performance which we are currently investigating.

## Conflicts of interest

There are no conflicts to declare.

## Supplementary Material

RA-008-C7RA13417G-s001
