# Supplementary material for: Water assisted atomic layer deposition of yttrium oxide using tris(N,N′-diisopropyl-2-dimethylamido-guanidinato) yttrium(iii): process development, film characterization and functional properties
Source: RSC Adv. 2018 Jan 29;8(9):4987–94. doi: 10.1039/c7ra13417g (PMC9078035; doi:10.1039/c7ra13417g)
Supplement: RA-008-C7RA13417G-s001 [file RA-008-C7RA13417G-s001.pdf]

Electronic Supplementary Material (ESI) for RSC Advances.
This journal is © The Royal Society of Chemistry 2018

Supporting Information

Water Assisted Atomic Layer Deposition of Yttrium Oxide Using Tris(*N*,*N'*-diisopropyl-2-dimethylamido-guanidinato) Yttrium(III): Process Development, Film Characterization and Functional Properties

Lukas Mai,[a†] Nils Boysen,[a†] Ersoy Subaşı,[b] Teresa de los Arcos,[c] Detlef Rogalla,[d] Guido Grundmeier,[c] Claudia Bock,[e] Hong-Liang Lu[f] and Anjana Devi[a*]

[a.] *Inorganic Materials Chemistry, Ruhr-University Bochum, 44801 Bochum, Germany. Email: anjana.devi@rub.de*
[b.] *Werkstoffe und Nanoelektronik, Ruhr-Universität Bochum, 44801 Bochum, Germany*
[c.] *Macromolecular and Technical Chemistry, University of Paderborn, 33098 Paderborn, Germany*
[d.] *RUBION, Ruhr-University Bochum, 44801 Bochum, Germany*
[e.] *Mikrosystemtechnik, Ruhr-Universität Bochum, 44801 Bochum. Germany*
[f.] *Institute of Advanced Nanodevices, School of Microelectronics, Fudan University, Shanghai 200433, China*

Synthesis of Tris(*N*,*N'*-diisopropyl-2-dimethylamido-guanidinato) Yttrium(III):

A suspension of $LiNMe_2$ (1.76 g, 34.5 mmol, Sigma-Aldrich, 95 % purity) in Et_2O (60 mL) was cooled to —20 °C. To this suspension, a mixture of *N*,*N*-diisopropylcarbodiimide (4.35 g, 34.5 mmol, Acros Organics, 99 % purity) and Et_2O (50 ml) were added dropwise and the mixture was allowed to warm up to room temperature and stirred for 24 h under argon atmosphere. The resulting pale-yellow solution of lithium diisopropyl guanidinate was transferred by a Teflon cannula in small portions to a suspension of YCl_3 (2.23 g, 11.45 mmol, ABCR, 99.9 % purity) in Et_2O (120 ml), which was cooled to 0 °C. The resulting mixture was allowed to warm to room temperature and stirring was continued for 48 h. The solvent was completely removed under vacuum, affording a slightly yellow solid. The crude product was extracted in hexane, which was then filtered through a Teflon cannula equipped with a Whatman filter to afford a pale-yellow solution. This solution was concentrated under vacuum. Spectroscopically pure colorless product in form of crystals were obtained from this concentrated solution after storage in a freezer at —20 °C overnight. Yield 4.82 g (70 %, based on YCl_3). Anal. Calc. for $C_{27}H_{60}N_9Y$ [%]: C, 54.07; H, 10.08; N, 21.02; Y, 14.82. Found [%]: C, 53.38; H, 10.58; N, 20.77. ^{1}H NMR (200 MHz, Benzene-d6) δ [ppm] = 3.60 (septd, J = 6.3, 1.7 Hz, 2H), 2.63 (s, 6H), 1.47 (d, J = 6.4 Hz, 7H), 1.29 (d, J = 6.2 Hz, 6H). ^{13}C NMR (50 MHz, C_6D_6) δ [ppm] = 172.32, 46.59, 40.33, 27.10, 26.61. EI- MS (70 eV): m/z (rel. int. [%]) = 599.52 (3.82 %, M+ = YL3+), 429.25 (13.93 %, YL2+), 385.2 (8.46 %, YL2+ - iPr), 258.09 (5.71 %, YL+). IR (ATR, [cm-1]): 2935.8 (m), 1433 (s), 1374.7 (s), 1363.3 (s), 1346.2 (m), 1323.5 (m), 1304.9 (m), 1172.6 (m), 1158.4 (m), 1125.7 (m), 1111.4 (m), 1033.2 (s), 443.9 (w), 688.8 (w), 728.6 (w).

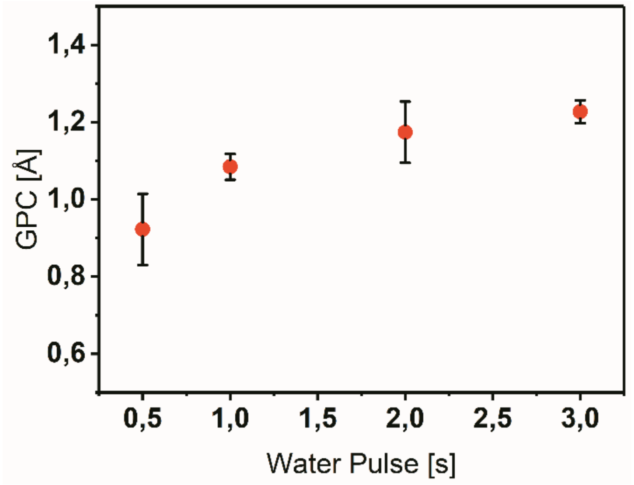

Figure S 1. The dependence between the water pulse times and GPC of the thin film at 225 °C.

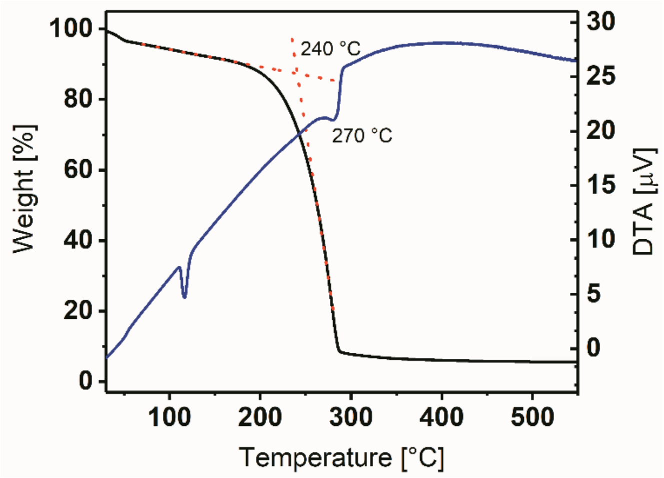

Figure S 2. Thermogravimetric (black curve) and differential thermal analysis (blue curve) for [Y(DPDMG)$_3$].

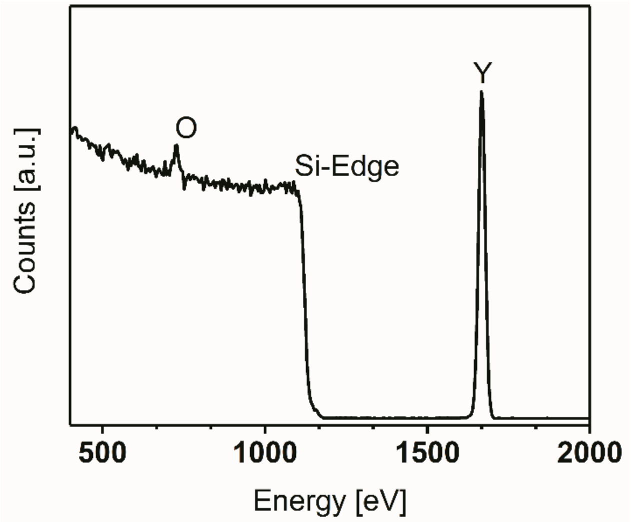

Figure S 3. RBS/NRA spectrum from a 30 nm Y_2O_3 thin film deposited on Si(100) at 225 °C.

Table S 1. Compositional analysis derived from XPS measurements for a 30 nm Y_2O_3 thin film deposited at 225 °C.

Analyzed Species	As introduced [at.%]	Sputtered [at.%]
Carbon	35.4	2.6
Nitrogen	0.3	--
Silicon	2.6	--
Yttrium	21.9	43.5
Oxygen	39.7	54.0
O/Y – Ratio	1.8	1.2

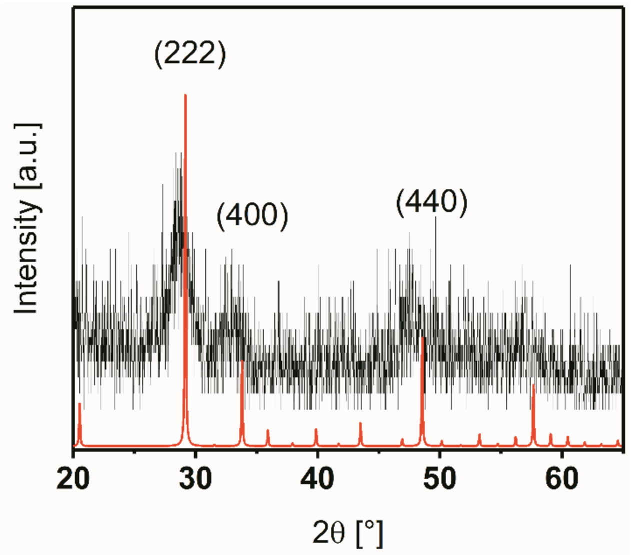

Figure S 4. GI-XRD of a 30 nm Y_2O_3 thin film deposited at 200 °C on Si(100).
The red pattern indicates the calculated powder XRD pattern of cubic Y_2O_3 (ICSD 185295).

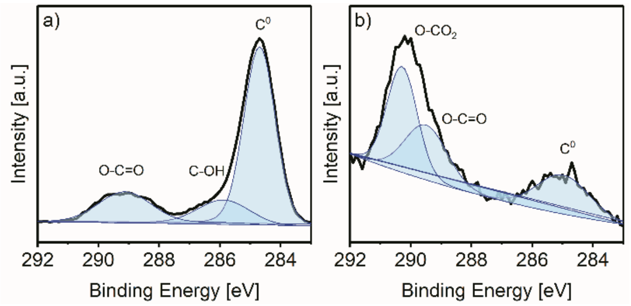

Figure S 5. a) Normalized XPS C1s core spectrum before sputtering. b) Normalized XPS C1s core spectrum after sputtering.
The black curve indicates the measured core, while the blue curve shows the fitted data.
